# Dimension-level network structure linking depression, anxiety, stress, sleep problems, and problematic smartphone use among chinese medical students

**DOI:** 10.3389/fpsyt.2026.1872025

**Published:** 2026-07-08

**Authors:** Wei Wu, Anping Liu, Sijie Gong

**Affiliations:** 1 Putian University, Putian, Fujian, China; 2 Fujian Medical University, Fuzhou, Fujian, China

**Keywords:** anxiety, depression, medical students, network analysis, problematic smartphone use, sleep problems, stress

## Abstract

**Background:**

Medical students experience converging risks of emotional distress, sleep disturbance, and problematic smartphone use, but the dimension-level conditional association patterns linking these domains remain insufficiently specified.

**Methods:**

This cross-sectional study surveyed 2,587 Chinese medical students (mean age = 18.88 ± 1.01 years; 55.51% female) using the 21-item DASS-21, the PSQI, and the MPAI. Regularized Gaussian graphical models were estimated with EBICglasso for the DASS-MPAI, PSQI-MPAI, and integrated DASS-PSQI-MPAI networks. Strength, bridge strength, node predictability, bootstrapped stability, and gender-based network differences were examined. Nodes represented DASS-21 dimensions, PSQI components, and MPAI dimensions rather than individual questionnaire items.

**Results:**

Anxiety was the most prevalent emotional distress dimension (39.89%), followed by depression (34.60%) and stress (15.58%). Sleep problems were detected in 23.42% of participants, whereas problematic smartphone use was detected in 64.71%. Across networks, nodes clustered into clearly differentiated emotional distress, sleep, and problematic smartphone use modules, with stronger within-domain than cross-domain edges. In the DASS-MPAI network, stress and withdrawal showed the highest strength, whereas depression and stress showed the highest bridge strength. In the PSQI-MPAI network, withdrawal and inefficiency were the strongest central nodes, and sleep disturbance and loss of control showed the highest bridge strength. In the integrated network, anxiety and stress showed the highest strength, followed by inefficiency and withdrawal. Bridge strength identified sleep disturbance (0.264), daytime dysfunction (0.239), and anxiety (0.235) as the most prominent cross-domain bridge nodes. Bootstrap analyses supported network stability; the integrated network centrality indices showed acceptable-to-good stability. Gender comparisons revealed no significant difference in global strength (P = 0.728), but the omnibus network structure test was significant (P = 0.010).

**Conclusions:**

This study provides a dimension-level map of conditional associations among emotional distress, sleep problems, and problematic smartphone use in a single-institution convenience sample of Chinese medical students. Anxiety, stress, sleep disturbance, daytime dysfunction, inefficiency, and withdrawal emerged as central or bridge nodes in the observed networks. These findings should be interpreted as exploratory cross-sectional associations rather than causal relationships or confirmed intervention targets, but they may inform hypotheses for future longitudinal and intervention studies.

## Introduction

Medical students are exposed to a multi-stressor educational environment characterized by intense academic competition, extensive coursework, clinical training demands, and high professional expectations. These pressures may contribute to emotional distress, impaired sleep, and maladaptive coping behaviors. Meta-analytic evidence indicates that depression and anxiety are common among Chinese medical students, and stress-related difficulties are also frequently reported (1). Sleep problems are another major concern in this population, because insufficient or poor-quality sleep may impair emotional regulation, daytime functioning, and academic performance (2, 3).

Problematic smartphone use (PSU), sometimes described in earlier literature as mobile phone addiction or smartphone addiction, has become a prominent behavioral issue among university students. In the present manuscript, PSU is used as the preferred term, while the Mobile Phone Addiction Index (MPAI) is retained as the name of the measurement instrument. PSU is generally characterized by impaired control over smartphone use, withdrawal-like experiences, escapist use, and reduced efficiency in daily activities. Previous reviews and empirical studies have linked PSU with depression, anxiety, stress, and poor sleep quality among adolescents and university students, including medical students (2, 4, 5).

Although prior studies have established associations between emotional distress, sleep problems, and PSU, most have examined these constructs using total scores or regression-based approaches. Such approaches are valuable for estimating overall associations but provide limited information about how specific dimensions or components are conditionally connected after other dimensions are controlled. From a network perspective, psychological and behavioral problems can be conceptualized as systems of mutually related components rather than as manifestations of a single latent cause (6, 7). Network analysis can identify nodes with high centrality or bridge strength within an observed conditional association network, thereby generating hypotheses about which dimensions or components may be important for future research.

However, interpretation of cross-sectional psychological networks requires caution. Partial-correlation networks identify conditional associations among measured variables, but they do not establish temporal ordering, causal pathways, mediation, or evidence that intervening on a central or bridge node will necessarily improve other nodes (8, 9). Therefore, centrality and bridge estimates in cross-sectional studies should be treated as exploratory markers of statistical connectivity. This distinction is especially important when the network is estimated at the dimension or component level rather than at the individual item level.

Existing network studies have explored the relationships among depression, anxiety, sleep problems, and smartphone use in college student populations (4, 10, 11). Nevertheless, several gaps remain. First, relatively few studies have simultaneously incorporated emotional distress, sleep problems, and PSU in a unified model among medical students. Second, many studies have not explicitly distinguished dimension-/component-level networks from fine-grained symptom-level networks. Third, institutional, cultural, and academic contexts may shape the configuration of these associations, and single-institution findings should be replicated in multicenter and cross-cultural samples.

To address these issues, this study surveyed 2,587 Chinese medical students and constructed three regularized partial-correlation networks: a DASS-MPAI network, a PSQI-MPAI network, and an integrated DASS-PSQI-MPAI network. The integrated network was considered the primary model, whereas the two-domain networks were estimated as secondary models to clarify how PSU was conditionally associated with emotional distress and sleep problems separately. The study aimed to: (1) characterize dimension-level conditional associations among emotional distress, sleep problems, and PSU; (2) identify central and bridge nodes in the observed networks; and (3) compare network structure between male and female students using an exploratory gender-based network comparison.

## Materials and methods

### Study design and participants

A cross-sectional questionnaire survey was conducted among undergraduate medical students at Putian University from November to December 2025. This study aimed to investigate the dimension-level network structure among depression, anxiety, stress, sleep problems, and PSU, and to further compare potential differences in the integrated network between male and female students.

Ethical approval for this study was obtained from the Ethics Review Committee of Putian University. Before accessing the questionnaire, all participants reviewed an electronic informed-consent statement and indicated consent before proceeding. Participation was voluntary and anonymous, and participants could withdraw from the survey at any time before submission.

The survey covered three domains. First, validated scales were used to assess depression, anxiety, and stress with the DASS-21, sleep problems with the PSQI, and PSU with the MPAI. Second, demographic characteristics were collected, including age, sex, residence, only-child status, height, and weight. Third, lifestyle-related characteristics were assessed, including smoking, electronic cigarette use, alcohol consumption, and coffee and tea intake.

The required sample size was informed by a simulation-based evaluation of network estimation performance (**Supplementary Figure 1**). The simulation was conducted for a 14-node integrated DASS-PSQI-MPAI network and evaluated network recovery across increasing case numbers using correlation, sensitivity, specificity, and centrality stability indices. The simulation suggested that a sample size of approximately 1,800 participants would provide adequate performance for the planned network estimation. A total of 2,616 questionnaires were collected. After data quality control, 2,587 valid responses were retained, yielding an effective response rate of 98.89%.

Data quality control was performed before analysis. Of the 2,616 submitted questionnaires, 29 were excluded because of obvious patterned responding or logical inconsistency. The final analyses were based on complete cases with valid DASS-21, PSQI, MPAI, and demographic data. No missing-value imputation was performed.

### Measures

**Supplementary Table 1** presents the mapping between network nodes and the corresponding scale dimensions or components. Because the present analyses used DASS-21 subscale scores, PSQI component scores, and MPAI dimension scores, the resulting networks are described as dimension-/component-level networks rather than individual symptom-level networks.

### Depression, anxiety, and stress

Depression, anxiety, and stress were assessed using the simplified Chinese version of the 21-item Depression Anxiety Stress Scales (DASS-21), originally developed by Lovibond and Lovibond and validated among Chinese college students by Gong et al. (12, 13). The DASS-21 consists of three seven-item subscales assessing depression, anxiety, and stress. Each item is rated on a 4-point scale from 0 to 3, with higher scores indicating greater emotional distress. Raw subscale scores were calculated by summing the seven items within each subscale. For descriptive prevalence estimates, raw subscale scores were multiplied by two to obtain DASS-42-equivalent scores. The following cut-offs were used to define mild-or-above emotional distress: depression >=10, anxiety >=8, and stress >=15. For network estimation, continuous DASS-21 subscale scores were used as dimension-level nodes rather than dichotomized variables: depressive symptoms (DASS1), anxiety symptoms (DASS2), and stress symptoms (DASS3).

### Sleep problems

Sleep problems were assessed using the Pittsburgh Sleep Quality Index (PSQI), which evaluates sleep status over the past month (14). The PSQI consists of seven components: subjective sleep quality, sleep latency, sleep duration, habitual sleep efficiency, sleep disturbance, use of sleep medication, and daytime dysfunction. These seven components were included as component-level nodes in the sleep domain, namely PSQI1-PSQI7. For descriptive reporting, a PSQI total score >7 was used to indicate the presence of sleep problems (15).

### Problematic smartphone use

Problematic smartphone use was assessed using the Mobile Phone Addiction Index (MPAI) (16). The MPAI consists of four dimensions: loss of control, withdrawal, escapism, and inefficiency. These four dimensions were included as continuous network nodes, namely MPAI1 (loss of control), MPAI2 (withdrawal), MPAI3 (escapism), and MPAI4 (inefficiency). For descriptive reporting, a total MPAI score >33 was used to indicate PSU according to the cut-off adopted in this study (17, 18).

### Statistical analysis

#### Descriptive analysis

Descriptive statistical analyses were conducted to summarize demographic characteristics, lifestyle variables, and the detection rates of depression, anxiety, stress, sleep problems, and PSU. Continuous variables were expressed as means and standard deviations, whereas categorical variables were presented as frequencies and percentages. All descriptive statistics were audited after data cleaning to ensure that frequencies and percentages were internally consistent.

#### Network estimation

Three networks were estimated: a DASS-MPAI two-domain network, a PSQI-MPAI two-domain network, and an integrated DASS-PSQI-MPAI three-domain network. The integrated network was considered the primary model because it jointly represented emotional distress, sleep problems, and PSU. The two-domain networks were estimated as secondary models to examine the conditional associations of PSU with emotional distress and sleep problems separately, thereby helping readers understand the incremental contribution of each domain before interpreting the integrated model.

Because all network nodes were summed or component scores treated as continuous indicators, Gaussian graphical models were used to estimate regularized partial correlations among nodes. EBICglasso was applied using the EBIC tuning parameter gamma = 0.50 to obtain sparse and interpretable networks (19). In the network graphs, nodes represented scale dimensions or components, whereas edges represented regularized partial correlations between two nodes after controlling for all other nodes in the network. Positive edges and negative edges were displayed using different line styles and colors, and thicker lines represented stronger associations. The bounded and skewed nature of several PSQI components is acknowledged as a methodological limitation.

#### Centrality, bridge centrality, and predictability

Strength centrality and bridge strength centrality were calculated for each node. Strength was defined as the sum of the absolute edge weights connected to a given node and was used to describe the overall statistical connectedness of each node within the observed network (20). Bridge strength was defined as the sum of the absolute edge weights connecting a given node to nodes in other predefined communities (21). Communities were predefined according to scale domains: DASS, PSQI, and MPAI. For visualization, prominent bridge nodes were selected according to the rank order of bridge strength. Specifically, the two highest-ranking bridge nodes were highlighted in each two-domain network, and the three highest-ranking bridge nodes were highlighted in the integrated network. These selections were descriptive and exploratory rather than based on causal or clinical thresholds. Node predictability was calculated and expressed as R2 to indicate the proportion of variance of a node explained by its neighboring nodes (22).

#### Stability and accuracy of the networks

Bootstrap procedures were used to evaluate the accuracy and stability of the network results. Nonparametric bootstrap analyses with 1,000 bootstrap samples were used to estimate edge-weight confidence intervals and to conduct difference tests for strength and bridge strength. Case-dropping bootstrap analyses were used to assess the stability of centrality indices under progressively reduced sample sizes, and the correlation stability coefficient was calculated. Values above 0.25 were considered acceptable and values above 0.50 were considered preferable.

#### Gender-based network comparison

The Network Comparison Test was used to compare the integrated DASS-PSQI-MPAI network between male and female students (23). Analyses were conducted with 1,000 permutations. Global strength and the omnibus network structure difference were tested. Edge-level differences were examined exploratorily with Benjamini-Hochberg correction where applicable. Because an omnibus network structure test does not by itself identify which specific edges differ reliably, gender-related interpretations were treated as exploratory unless supported by corrected edge-level results.

All statistical analyses were performed in R software, version 4.3.2. Network analyses were primarily conducted using the qgraph, bootnet, networktools, mgm, and NetworkComparisonTest packages.

## Results

### Basic characteristics of participants and detection rates of depression, anxiety, stress, sleep problems, and PSU

The demographic characteristics of the study participants are presented in **Table 1**. A total of 2,587 participants were included in this study, with a mean age of 18.88 ± 1.01 years and a mean BMI of 22.17 ± 4.94 kg/m2. The proportion of female participants was slightly higher than that of male participants, with 1,436 females accounting for 55.51% of the sample and 1,151 males accounting for 44.49%. Most participants were from rural areas (73.06%), and the majority were not only children (80.36%).

**Table 1:** Sociodemographic characteristics, health-related behaviors, and prevalence of depression, anxiety, stress, sleep problems, and problematic smartphone use in the study participants.

Variables	Mean/N	SD/%
Age, years	18.88	1.01
Height, cm	167.90	9.09
Weight, kg	62.85	16.13
BMI, kg/m2	22.17	4.94
Gender		
Male	1151	44.49
Female	1436	55.51
Residence		
Urban	697	26.94
Rural	1890	73.06
Family sibling status		
Only child	508	19.64
Non-only child	2079	80.36
Smoking		
Rarely or never	2335	90.26
Occasionally	99	3.83
Frequently or almost every day	153	5.91
E-cigarette use		
Rarely or never	2462	95.17
Occasionally	30	1.16
Frequently or almost every day	95	3.67
Alcohol consumption		
Rarely or never	2273	87.86
Occasionally	30	1.16
Frequently or almost every day	284	10.98
Coffee consumption		
Rarely or never	1713	66.22
Occasionally	106	4.10
Frequently or almost every day	768	29.69
Tea consumption		
Rarely or never	1410	54.50
Occasionally	146	5.64
Frequently or almost every day	1031	39.85
Depression		
No depression	1692	65.40
With depression	895	34.60
Anxiety		
No anxiety	1555	60.11
With anxiety	1032	39.89
Stress		
No stress	2184	84.42
With stress	403	15.58
Sleep problems		
Normal sleep (PSQI 0-7)	1981	76.58
Have sleep problems (PSQI 8-21)	606	23.42
Problematic smartphone use		
No problematic smartphone use	913	35.29
With problematic smartphone use	1674	64.71

Regarding lifestyle characteristics, most participants reported rarely or never smoking (90.26%), using electronic cigarettes (95.17%), or consuming alcohol (87.86%). Coffee and tea intake were relatively more common, with 29.69% and 39.85% of participants reporting frequent or almost daily consumption of coffee and tea, respectively.

The detection rates of emotional distress dimensions showed that anxiety was the most common, affecting 39.89% of participants (n = 1,032), followed by depression, which was detected in 34.60% of participants (n = 895). Stress was detected in 15.58% of participants (n = 403). Sleep problems, defined as a PSQI total score >7, were detected in 23.42% of participants (n = 606). In addition, PSU, defined as an MPAI total score >33, was detected in 64.71% of participants (n = 1,674). **Supplementary Tables 2a–c** summarize distributional characteristics, centrality indices, bridge strength, and predictability for the three network models.

### DASS-MPAI network and bridge nodes

**Figure 1:**
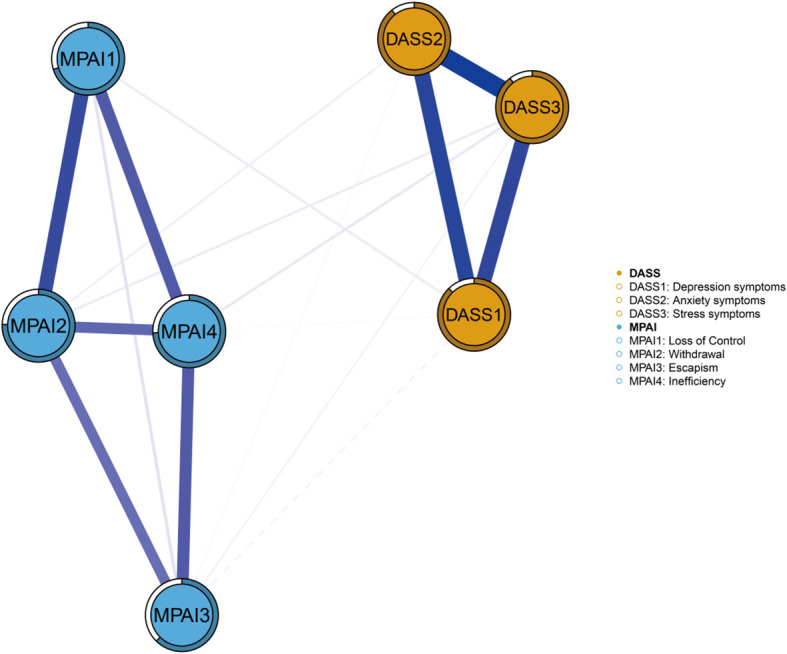
Dimension-level network structure of DASS and MPAI dimensions among the study participants. Orange nodes represent DASS dimensions, including depression, anxiety, and stress; blue nodes represent MPAI dimensions, including loss of control, withdrawal, escapism, and inefficiency. Edges indicate regularized partial correlations between nodes after controlling for all other variables. Thicker edges indicate stronger associations.

**Figure 1** presents the dimension-level network of DASS and MPAI nodes. The network exhibited a relatively clear two-module structure, with the emotional distress domain and the PSU domain each forming a cohesive cluster. Positive associations predominated across the network, and stronger edges were mainly concentrated within each domain. This pattern suggests that emotional distress and PSU dimensions were conditionally associated but retained distinguishable structural characteristics.

**Figure 2:**
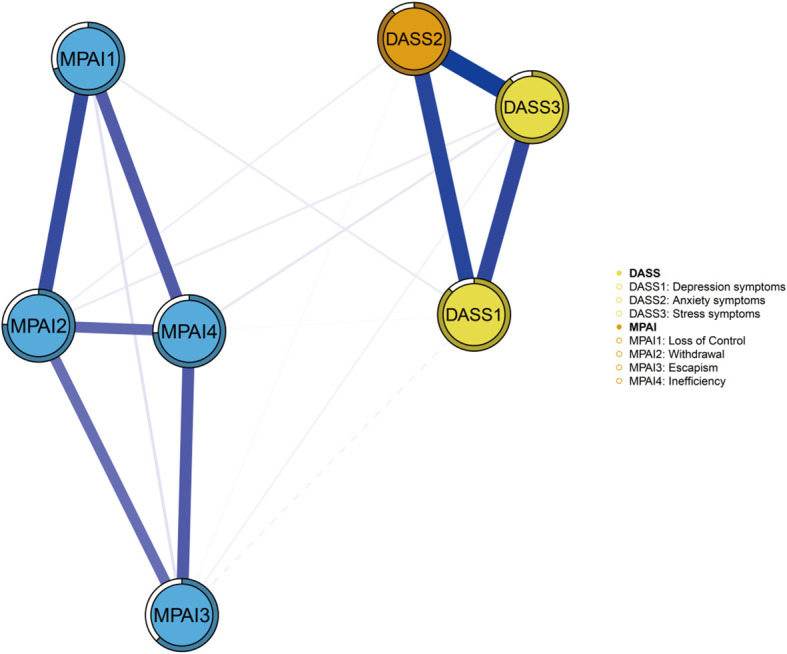
Bridge nodes within the DASS-MPAI network among the study participants. Yellow nodes indicate the two highest-ranking bridge nodes identified on the basis of bridge strength centrality. Bridge-node identification was exploratory and based on the rank order of bridge strength.

In terms of strength centrality, stress (DASS3 = 1.063) and withdrawal (MPAI2 = 1.056) ranked highest, followed by inefficiency (MPAI4 = 1.018) and anxiety (DASS2 = 1.011). These nodes were more strongly connected to the rest of the DASS-MPAI network. By contrast, escapism (MPAI3 = 0.735) had the lowest strength centrality. Bridge strength analysis identified stress (DASS3 = 0.105) and depression (DASS1 = 0.068) as the two highest-ranking bridge nodes linking emotional distress and PSU in this two-domain network. Node predictability values were relatively high for the DASS dimensions, with R2 values of 0.879 for depression, 0.895 for anxiety, and 0.896 for stress (**Figure 2**).

The stability and accuracy analyses of the DASS-MPAI network are presented in **Supplementary Figure 3**. Bootstrap difference tests indicated that several differences in strength and bridge strength were statistically distinguishable. Case-dropping bootstrap analyses suggested acceptable stability of centrality indices, and bootstrapped edge-weight confidence intervals supported the precision of the major edge-weight estimates.

### PSQI-MPAI network and bridge nodes

**Figure 3:**
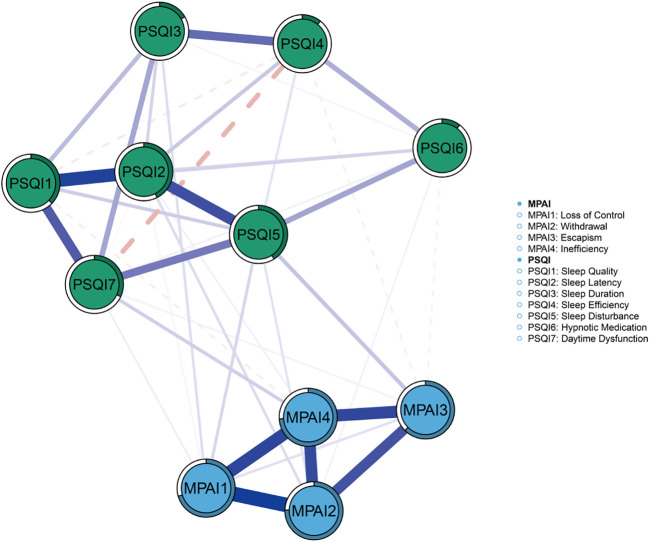
Component-/dimension-level network structure of PSQI and MPAI dimensions among the study participants. Green nodes represent PSQI components, including sleep quality, sleep latency, sleep duration, sleep efficiency, sleep disturbance, hypnotic medication use, and daytime dysfunction; blue nodes represent MPAI dimensions.

**Figure 3** illustrates the component-/dimension-level network of PSQI and MPAI nodes. Similar to the DASS-MPAI network, the PSQI-MPAI network exhibited two relatively compact modules corresponding to sleep problems and PSU. Within-domain edges were generally stronger than cross-domain edges, suggesting that the direct conditional associations between sleep and PSU were concentrated in a limited number of nodes rather than distributed evenly across all components.

**Figure 4:**
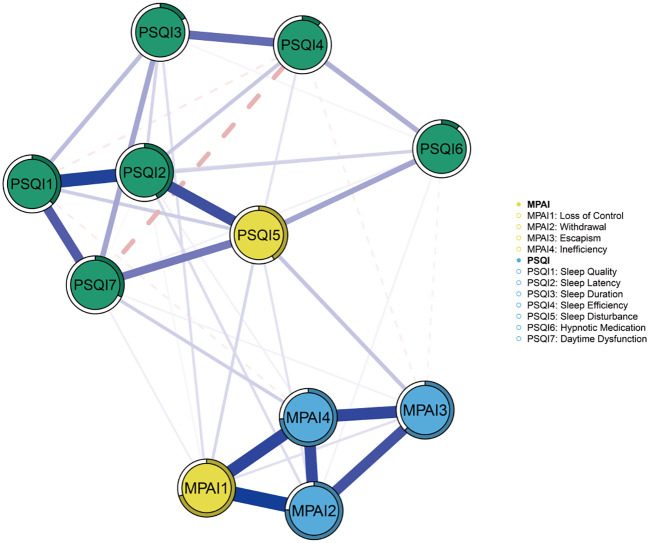
Bridge nodes within the PSQI-MPAI network among the study participants. Yellow nodes indicate the two highest-ranking bridge nodes identified on the basis of bridge strength centrality. Bridge-node identification was exploratory and descriptive.

In terms of strength centrality, withdrawal (MPAI2 = 1.125) and inefficiency (MPAI4 = 1.109) ranked highest, followed by sleep latency (PSQI2 = 0.957), loss of control (MPAI1 = 0.951), and sleep disturbance (PSQI5 = 0.949). Bridge strength analysis indicated that sleep disturbance (PSQI5 = 0.189) and loss of control (MPAI1 = 0.156) were the two highest-ranking bridge nodes linking sleep problems and PSU. These estimates should be interpreted as cross-sectional conditional associations rather than evidence that these nodes causally transmit effects between domains (**Figure 4**).

The stability and accuracy analyses of the PSQI-MPAI network are presented in **Supplementary Figure 5**. The bootstrap analyses supported the stability of the main centrality and bridge centrality findings, although the bounded distributions of several PSQI components should be considered when interpreting the results.

### Integrated DASS-PSQI-MPAI network

**Figure 5:**
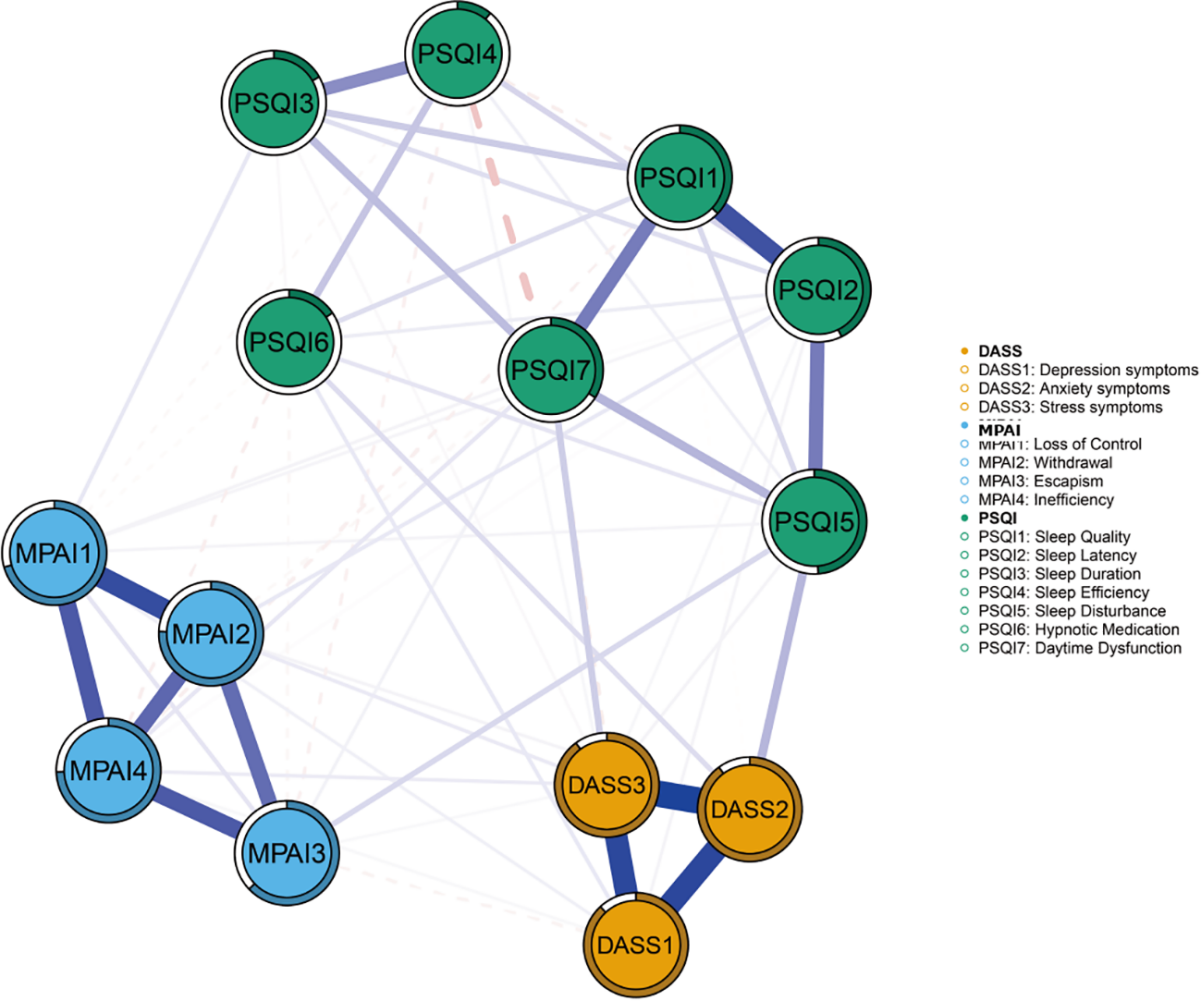
Integrated dimension-/component-level network structure of DASS, PSQI, and MPAI dimensions among the study participants. Orange nodes represent DASS dimensions; green nodes represent PSQI components; blue nodes represent MPAI dimensions. Edges indicate regularized partial correlations between nodes after controlling for all other variables.

**Figure 5** presents the integrated dimension-/component-level network comprising DASS dimensions, PSQI components, and MPAI dimensions. Overall, positive associations predominated across the network, and stronger edges were mainly concentrated within each domain. Cross-domain edges were relatively sparse and weaker, indicating that direct connections among emotional distress, sleep problems, and PSU were maintained primarily by a limited number of nodes.

**Figure 6:**
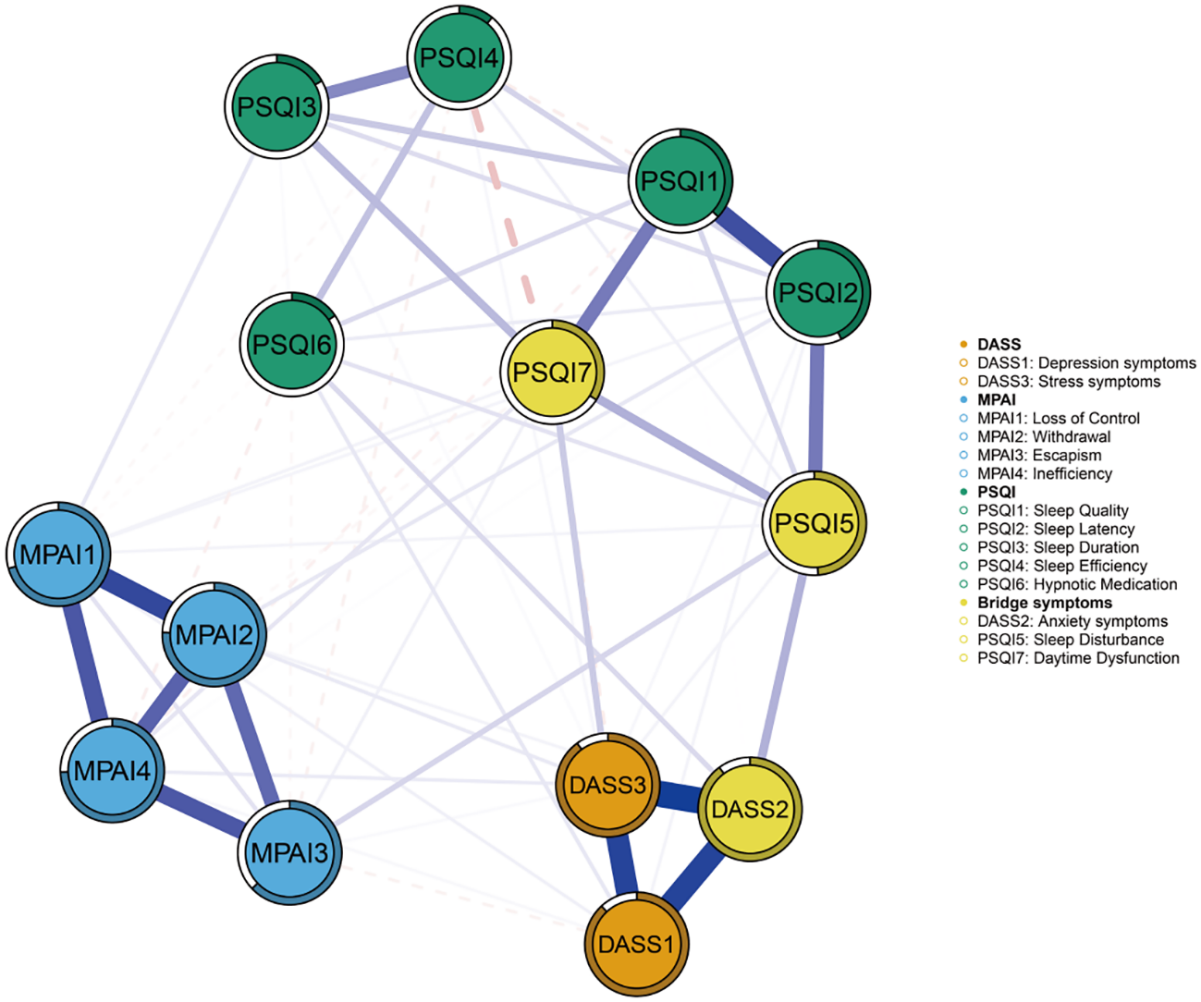
Bridge nodes within the integrated DASS-PSQI-MPAI network among the study participants. Yellow nodes indicate the three highest-ranking bridge nodes identified on the basis of bridge strength centrality: anxiety, sleep disturbance, and daytime dysfunction.

Centrality analysis showed that anxiety (DASS2 = 1.175) and stress (DASS3 = 1.164) had the highest strength values, followed by inefficiency (MPAI4 = 1.118) and withdrawal (MPAI2 = 1.101). Bridge strength analysis identified sleep disturbance (PSQI5 = 0.264), daytime dysfunction (PSQI7 = 0.239), and anxiety (DASS2 = 0.235) as the three highest-ranking cross-domain bridge nodes. These findings suggest that selected sleep and emotional distress nodes were statistically important cross-domain connectors in the observed network, but they do not establish temporal or causal pathways (**Figure 6**).

The stability and accuracy of the integrated DASS-PSQI-MPAI network are presented in **Supplementary Figure 8**. Based on 1,000 bootstrap samples, centrality and bridge centrality results showed acceptable-to-good stability. In addition, bootstrapped 95% confidence intervals for edge weights supported the reproducibility of the major edge estimates in the network.

### Gender-based comparison of the integrated network

**Figure 7:**
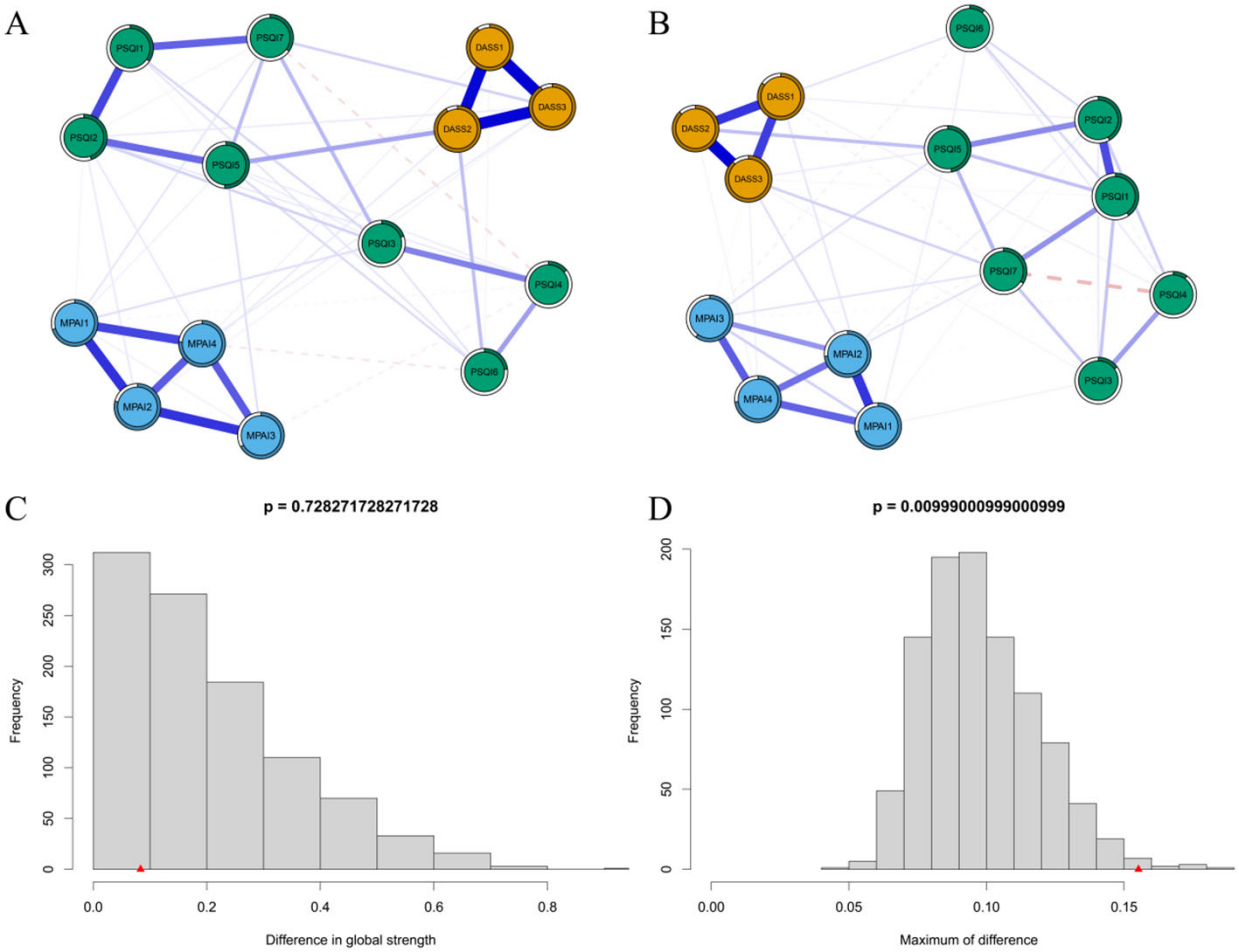
Comparative network analysis of DASS, PSQI, and MPAI dimensions by gender among the study participants. **(A)** Network structure for male participants. **(B)** Network structure for female participants. **(C)** Permutation-based distribution of differences in global strength between genders, indicating no significant difference in overall connectivity (P = 0.728). **(D)** Permutation-based distribution of the maximum network structure difference, indicating a significant omnibus gender difference in network structure (P = 0.010).

**Figure 7** presents the gender-based comparison of the integrated DASS-PSQI-MPAI network. Regarding global strength, no significant difference was observed between males and females (P = 0.728), indicating that the overall connectivity of the network was comparable between the two groups. However, the omnibus network structure comparison showed a significant difference between male and female networks (P = 0.010). Because the omnibus test does not by itself identify which specific edges differ reliably, and because gender was measured only as a binary demographic variable, these findings should be interpreted cautiously and treated as exploratory.

## Discussion

### Main findings

This study used regularized partial-correlation network analysis to examine dimension-level conditional associations among emotional distress, sleep problems, and PSU in a large sample of Chinese medical students. The networks showed stronger within-domain than cross-domain connectivity, suggesting that DASS dimensions, PSQI components, and MPAI dimensions formed relatively distinct but interrelated modules. In the integrated network, anxiety, stress, sleep disturbance, daytime dysfunction, inefficiency, and withdrawal showed relatively high strength or bridge strength. These findings should be interpreted as exploratory indicators of statistical connectivity in the observed cross-sectional networks rather than evidence of causal pathways or confirmed intervention targets.

The DASS-MPAI network indicated that stress and withdrawal had the highest strength centrality, whereas depression and stress showed the highest bridge strength between emotional distress and PSU in the two-domain network. This pattern suggests that stress-related and depressive dimensions may be particularly relevant for understanding the conditional associations between emotional distress and PSU among medical students. However, because the present design was cross-sectional, these findings do not demonstrate that emotional distress leads to PSU or that PSU causes emotional distress.

The PSQI-MPAI network highlighted withdrawal and inefficiency as the most central PSU dimensions and identified sleep disturbance and loss of control as the highest-ranking bridge nodes between sleep problems and PSU. In the integrated network, sleep disturbance and daytime dysfunction remained important cross-domain bridge nodes, and anxiety also showed high bridge strength. These findings are consistent with previous evidence that poor sleep, emotional distress, and problematic smartphone-related behaviors are interrelated among university students (2, 4, 10, 24, 25). The current results extend this literature by showing that these associations are not evenly distributed across all dimensions or components, but are concentrated in selected nodes.

The observed role of sleep disturbance and daytime dysfunction is particularly noteworthy. Sleep disturbance may reflect difficulties maintaining sleep or interruptions during the night, whereas daytime dysfunction captures impaired wake-time functioning. Both components may be closely linked to academic functioning, emotional regulation, and technology-related behaviors among medical students. Nevertheless, the present network cannot test whether sleep disturbance mediates the relationship between emotional distress and PSU, nor can it determine whether sleep-related interventions would produce broader changes in the network. These questions require longitudinal, experimental, or experience-sampling designs.

The gender-based Network Comparison Test indicated comparable global strength but a significant omnibus difference in network structure between male and female students. This finding suggests that the overall degree of connectedness was similar across groups, whereas some local association patterns may differ. However, the present study measured sex/gender only as a binary demographic variable and did not assess psychological mechanisms that might explain gender-related differences, such as loneliness, impulsivity, social motivation, or emotion-regulation strategies. Therefore, gender-specific interpretations should be considered exploratory and require replication with corrected edge-level analyses and richer psychological measurements.

Prior theoretical and empirical literature suggests several plausible mechanisms linking emotional distress, smartphone use, and sleep problems, including psychological hyperarousal, nighttime screen exposure, time displacement, self-control difficulties, and impaired daytime functioning (26–30). The present findings are compatible with these hypotheses but cannot directly test them. Similarly, psychological resilience, cognitive emotion regulation, coping style, and self-control may influence the association between emotional distress and PSU (31–34), but these variables were not measured in the current study. They should therefore be treated as hypotheses for future research rather than explanations supported by the present data.

The present findings may help generate hypotheses for campus-based mental health and digital-health interventions. For example, stress-management programs, sleep-hygiene education, and healthier smartphone-use practices could be evaluated in longitudinal or randomized designs to determine whether modifying highly connected nodes leads to broader improvements. As a conceptual extension, interventions may also consider peer-norm messaging, such as communicating that balanced smartphone use, reduced nighttime use, and adherence to sleep-hygiene practices are common and valued among fellow students. This norm-based logic has been discussed in public-health behavior research (35), but it should be regarded as a conceptual extension rather than direct evidence from the present network.

### Strengths and limitations

This study has several strengths. First, it included a large sample of more than 2,500 medical students, providing a stable basis for estimating a relatively small network. Second, the study jointly examined emotional distress, sleep problems, and PSU in an integrated model while also presenting two secondary two-domain networks. Third, predictability, bootstrap stability, and gender-based network comparison were reported, improving the transparency of the network analysis.

Several limitations should also be acknowledged. First, the cross-sectional design only allows identification of conditional associations among nodes and does not permit causal inference. Centrality and bridge strength do not establish temporal precedence, causal maintenance, mediation, or confirmed intervention targets. Future longitudinal and experience-sampling studies are needed to examine dynamic temporal relationships among emotional distress, sleep problems, and PSU.

Second, the sample was a convenience sample drawn from a single university in China, which may limit generalizability. Institutional culture, academic schedules, local norms around smartphone use, and sociocultural context may influence the observed network structure. Multicenter and cross-cultural studies are needed to evaluate whether the present findings replicate across different institutions and student populations.

Third, the present network was estimated at the dimension/component level rather than the item level. Aggregating seven DASS-21 items into one dimension score may reduce item-level heterogeneity, decrease the number of within-domain edges, and affect the comparability of centrality and bridge-strength estimates across domains. The node granularity also differed across domains, with DASS represented by three subscale scores, PSQI by seven component scores, and MPAI by four dimension scores. Therefore, the results should not be interpreted as a fine-grained symptom-level map. Future item-level networks could identify more precise intervention-relevant symptoms.

Fourth, demographic and lifestyle variables such as BMI, residence, smoking, alcohol use, caffeine intake, and tea consumption were collected but were not included as nodes or covariates in the primary network models. These factors may influence emotional distress, sleep quality, and PSU. Future studies should evaluate covariate-adjusted, stratified, or moderated network models.

Fifth, all measures were self-reported. Objective indicators such as smartphone use logs, screen time, wearable sleep monitoring data, and physiological stress indicators were not available. Finally, psychological resilience, emotion regulation, loneliness, impulsivity, perceived peer norms, and coping strategies were not measured, limiting the ability to interpret the mechanisms underlying the observed network associations.

### Future research

Future research should adopt longitudinal designs or experience-sampling methods to collect repeated assessments of emotional distress, sleep, and smartphone use, and should employ temporal or dynamic network models to examine the directionality of associations (36, 37). Objective indicators, such as smartphone usage logs, wearable sleep data, and heart rate variability, may help validate the network structure from behavioral and physiological perspectives.

Cross-cultural and cross-disciplinary studies are also needed to determine whether the observed network characteristics are specific to medical students or generalize to other student populations. In addition, future work should incorporate protective and contextual variables, such as psychological resilience, cognitive emotion regulation, perceived peer norms, social support, and academic stress, to construct more comprehensive risk-resilience network models.

## Conclusion

This study used network analysis to examine dimension-level conditional associations among depression, anxiety, stress, sleep problems, and PSU among Chinese medical students. The observed networks were characterized by stronger within-domain than cross-domain connectivity. In the integrated network, anxiety, stress, inefficiency, withdrawal, sleep disturbance, and daytime dysfunction emerged as central or bridge nodes. Global network strength did not differ significantly by gender, but the omnibus network structure test was significant. These findings should be interpreted as exploratory cross-sectional associations rather than causal relationships. Future longitudinal, multicenter, and intervention studies are needed to validate these network characteristics and determine whether modifying highly connected nodes produces broader improvements in mental health, sleep, and smartphone-use outcomes.

## Data Availability

The raw data supporting the conclusions of this article will be made available by the authors, without undue reservation.
