# Supplementary material for: Dimension-level network structure linking depression, anxiety, stress, sleep problems, and problematic smartphone use among chinese medical students
Source: Front Psychiatry. 2026 Jul 8;17:1872025. doi: 10.3389/fpsyt.2026.1872025 (PMC13388836; doi:10.3389/fpsyt.2026.1872025)
Supplement: Supplementary file 1 [file SupplementaryFile1.docx]

**Supplementary material**


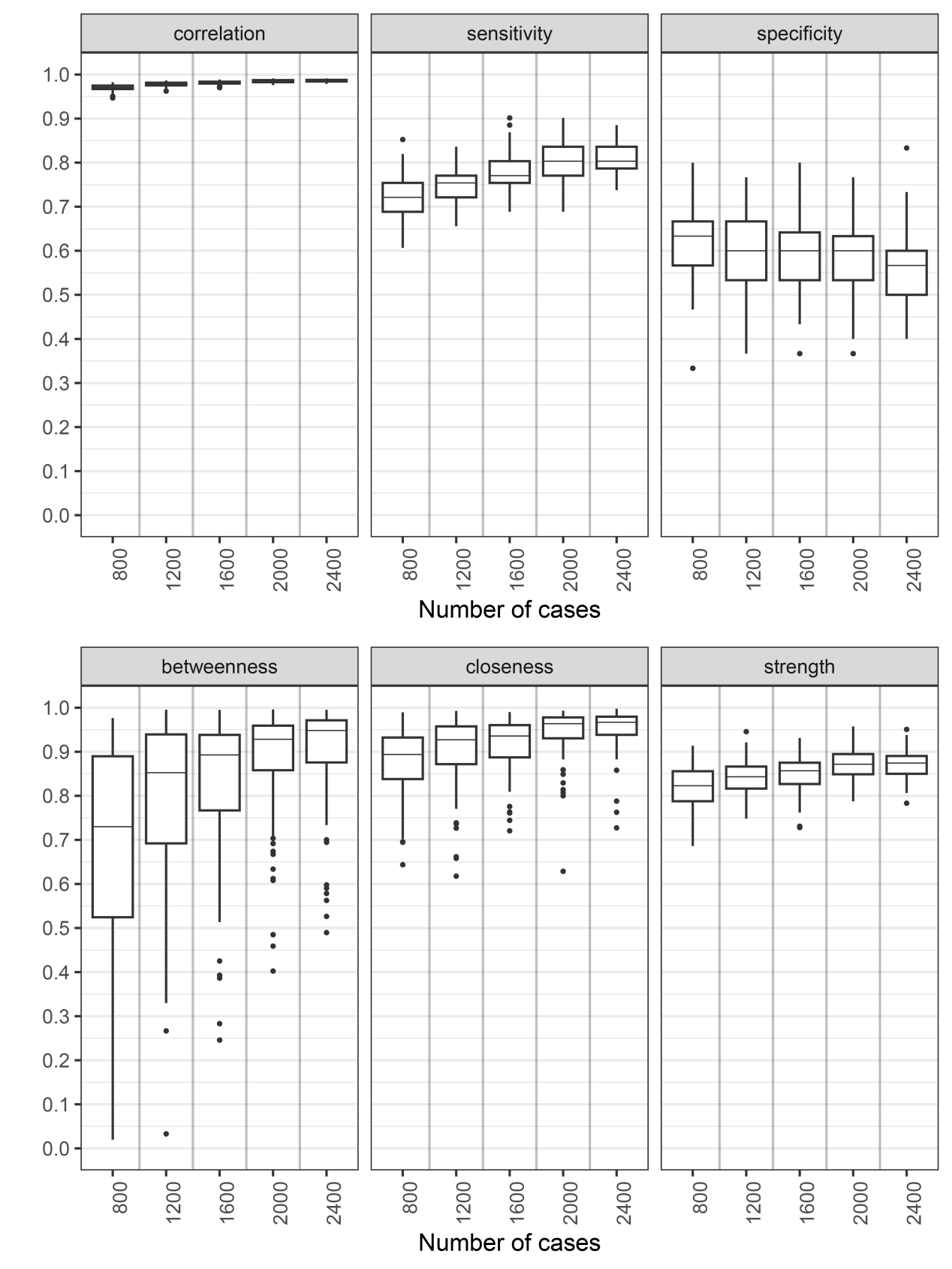


**Figure S1.** Simulation-based sample-size evaluation for the integrated DASS-PSQI-MPAI dimension-/component-level network among the study participants. The upper panels present network recovery indices across increasing sample sizes, including correlation, sensitivity, and specificity. The lower panels present the stability of betweenness, closeness, and strength centrality indices across case numbers.


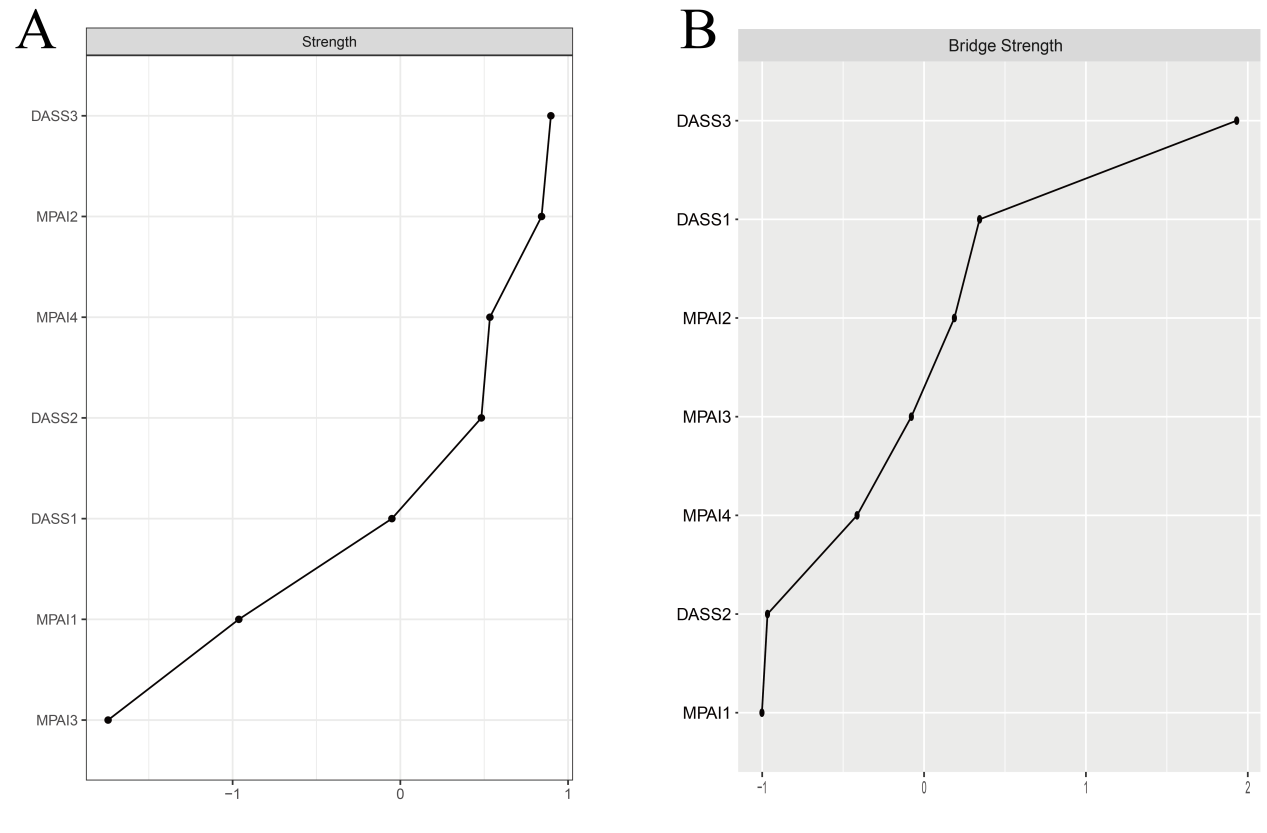


**Figure S2.** Standardized strength and bridge strength centrality indices for the DASS-MPAI dimension-level network among the study participants (z-scores). (A) Strength centrality; (B) bridge strength centrality.

**
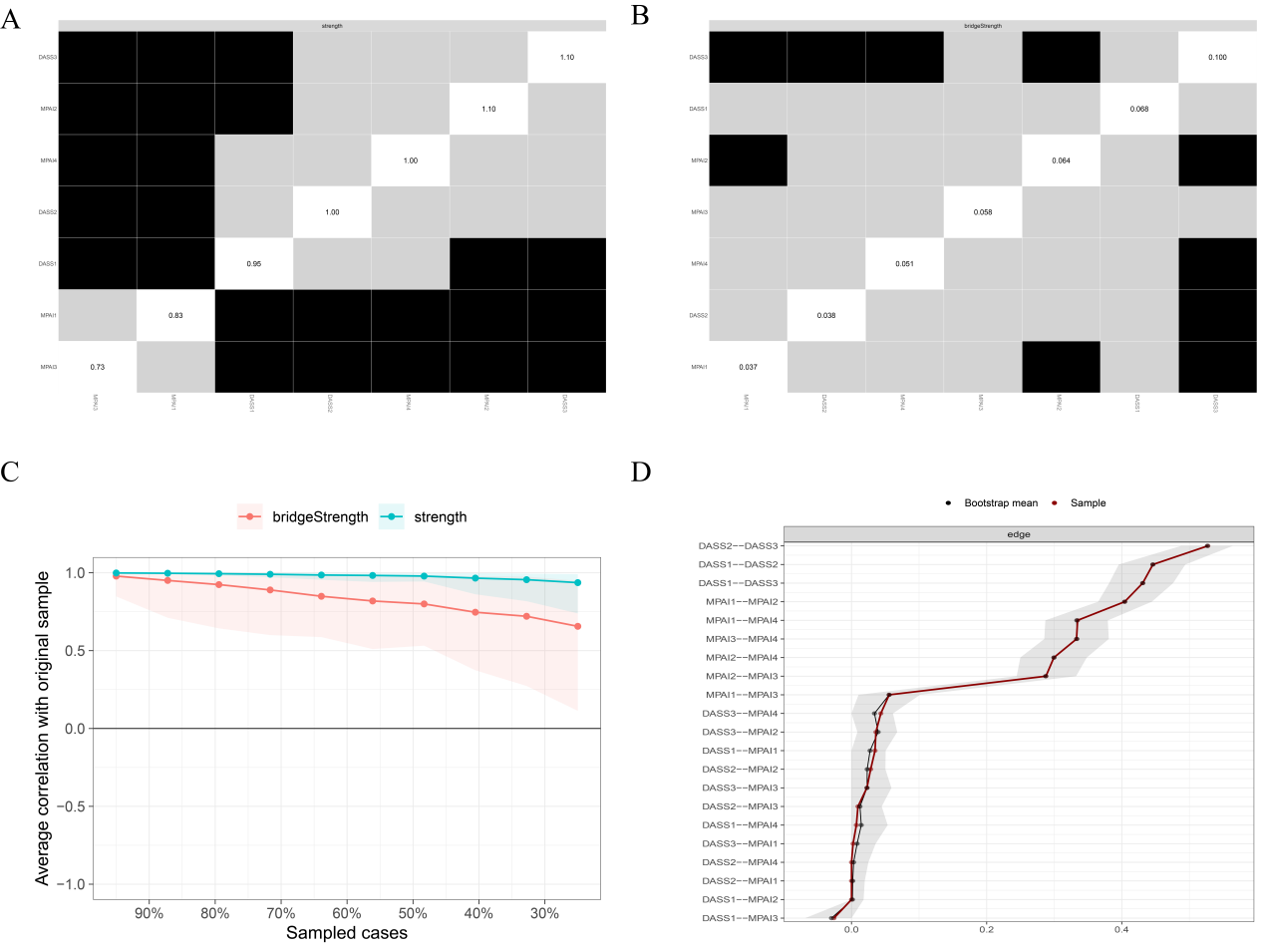
**

**Figure S3. Stability and accuracy assessment of the DASS-MPAI dimension-level network among the study participants.**

(A) Bootstrapped difference tests (1,000 iterations) for nodal strength centrality. (B) Nonparametric bootstrapped difference tests for bridge strength centrality. Gray cells indicate non-significant differences (α = 0.05), black cells indicate significant differences, and diagonal white cells display the original sample estimates. (C) Case-dropping bootstrap analysis of the stability of strength and bridge strength centrality. The x-axis represents the proportion of cases retained in the re-estimated networks, and the y-axis shows the average correlation between centrality indices in the original and subset networks. The solid line denotes strength and the dashed line denotes bridge strength; CS coefficients ≥0.50 were considered to indicate acceptable stability. (D) Bootstrapped 95% confidence intervals for edge weights. Red lines represent sample estimates, and gray shaded areas indicate the 1,000-bootstrap 95% confidence intervals. Edge labels are presented along the y-axis.


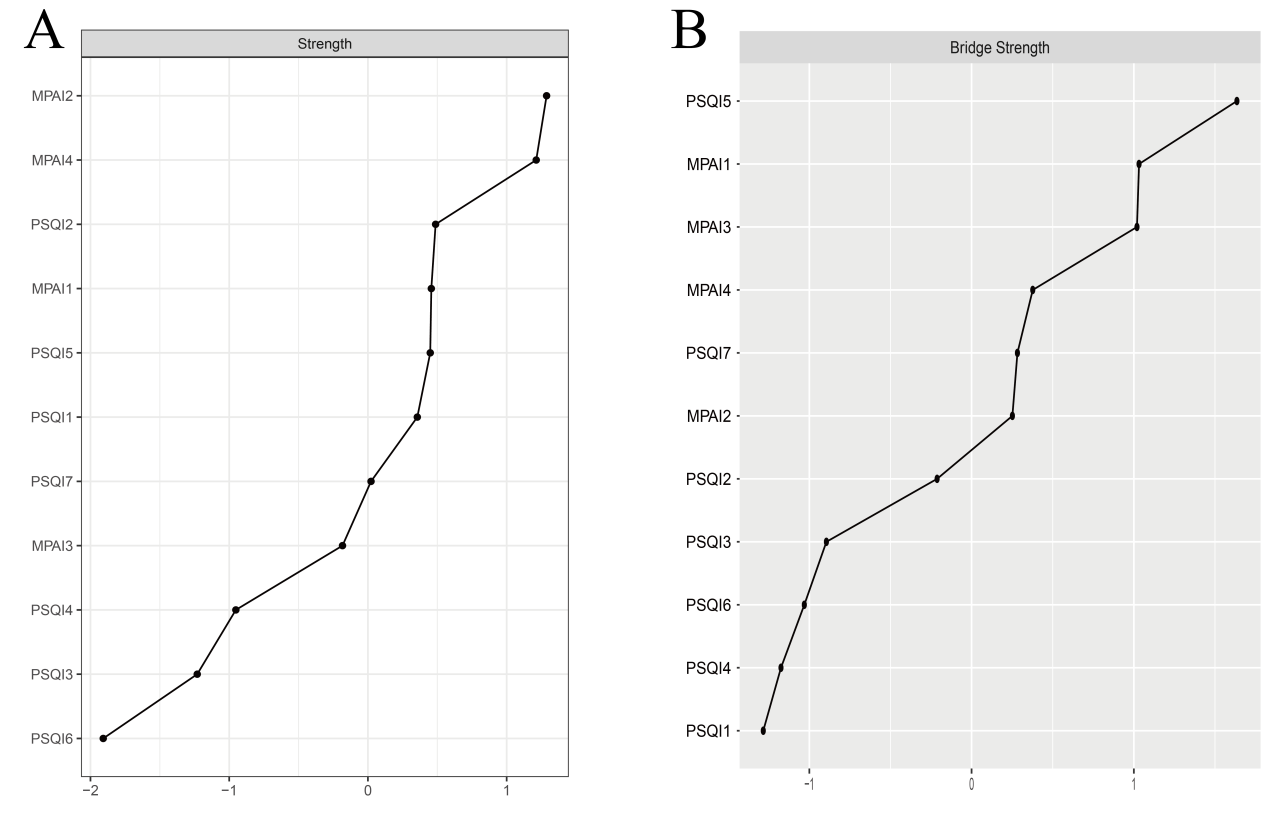


**Figure S4.** Standardized strength and bridge strength centrality indices for the PSQI-MPAI component-/dimension-level network among the study participants (z-scores). (A) Strength centrality; (B) bridge strength centrality.


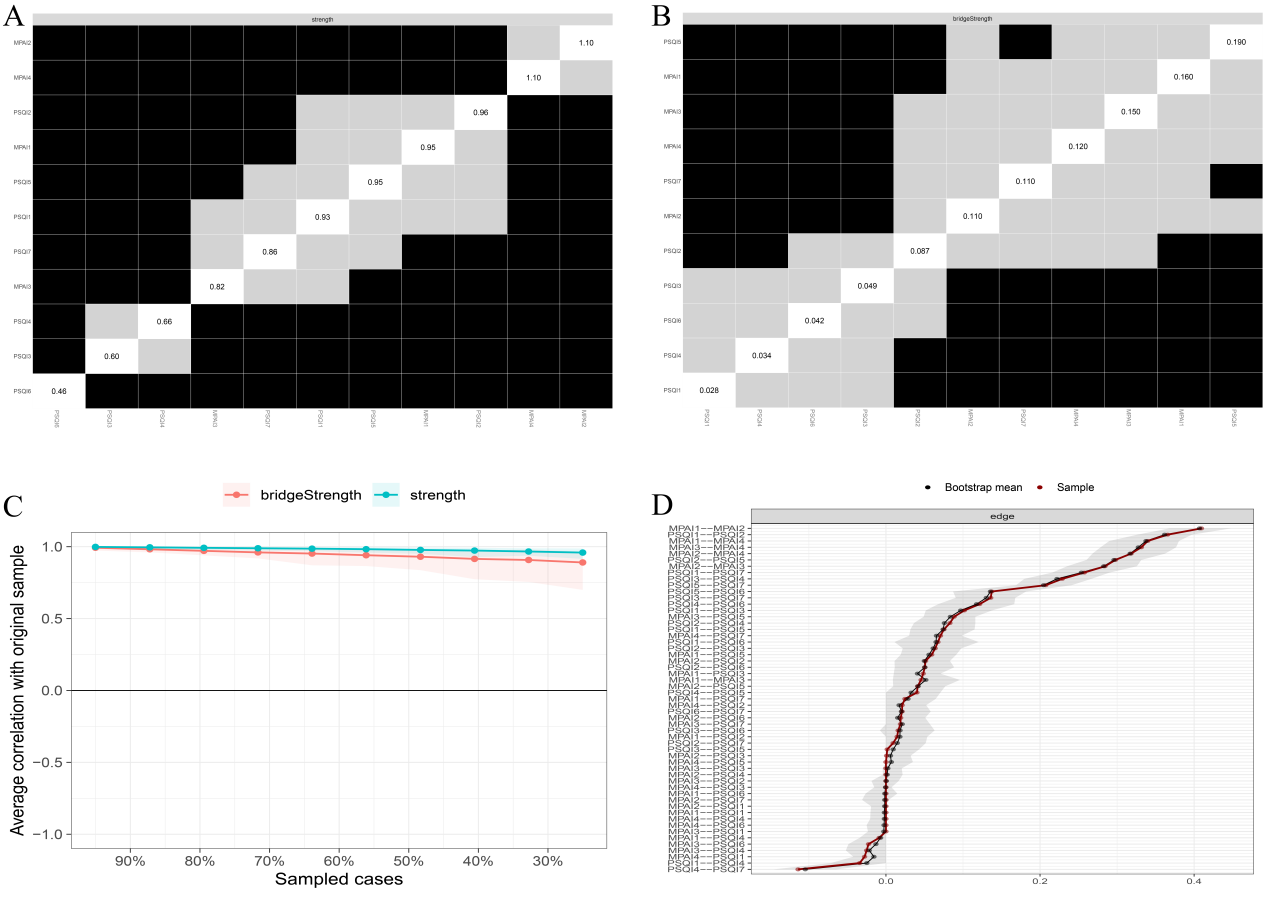


**Figure S5.** Stability and accuracy assessment of the PSQI-MPAI component-/dimension-level network among the study participants. (A) Bootstrap difference tests for nodal strength centrality. (B) Bootstrap difference tests for bridge strength centrality. Gray cells indicate non-significant differences, black cells indicate significant differences, and diagonal white cells display the original sample estimates. (C) Case-dropping bootstrap analysis of the stability of strength and bridge strength centrality. (D) Bootstrapped 95% confidence intervals for edge weights. All bootstrap analyses were based on 1,000 bootstrap samples.


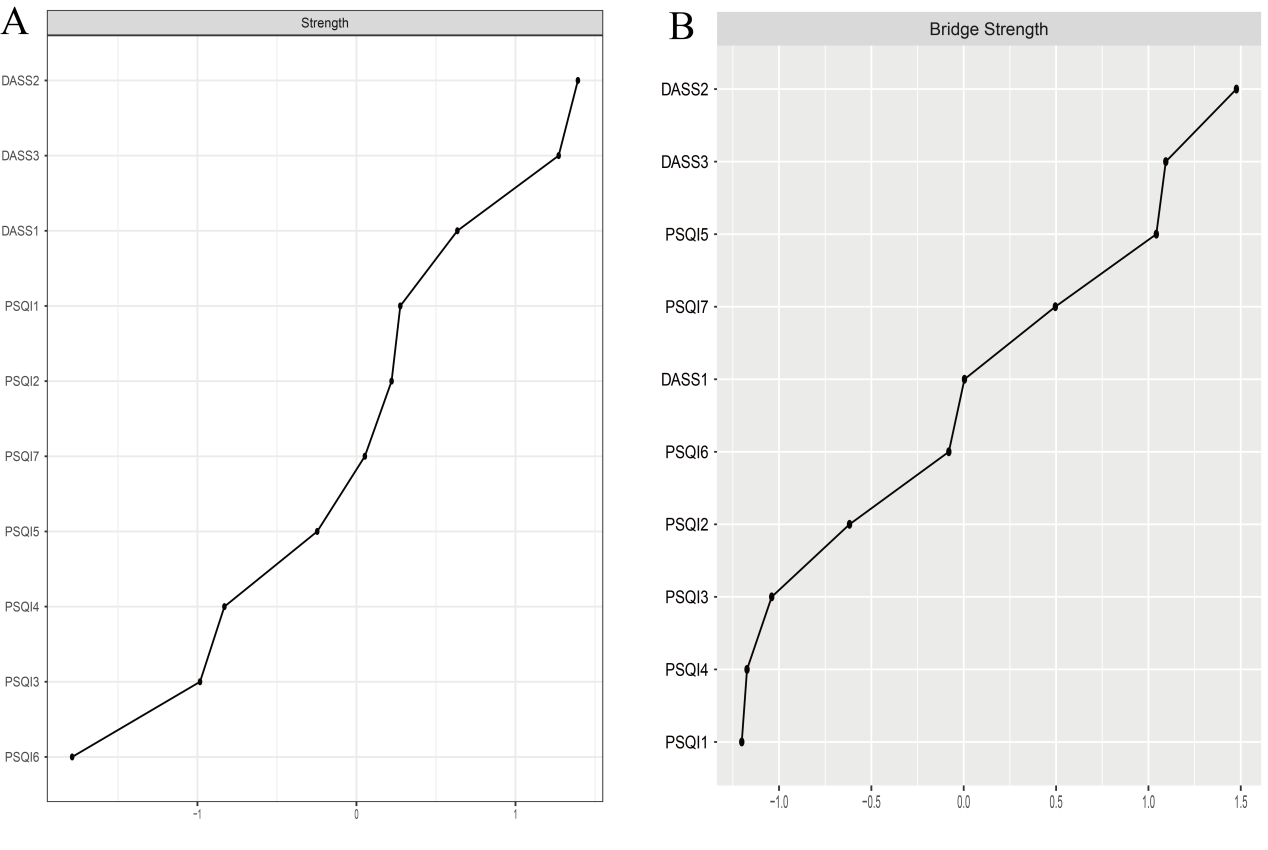


**Figure S6.** Exploratory standardized strength and bridge strength centrality indices for the DASS-PSQI dimension-/component-level network among the study participants (z-scores). This exploratory two-domain network is provided only as supplementary information and was not the primary model in the revised manuscript. (A) Strength centrality; (B) bridge strength centrality.


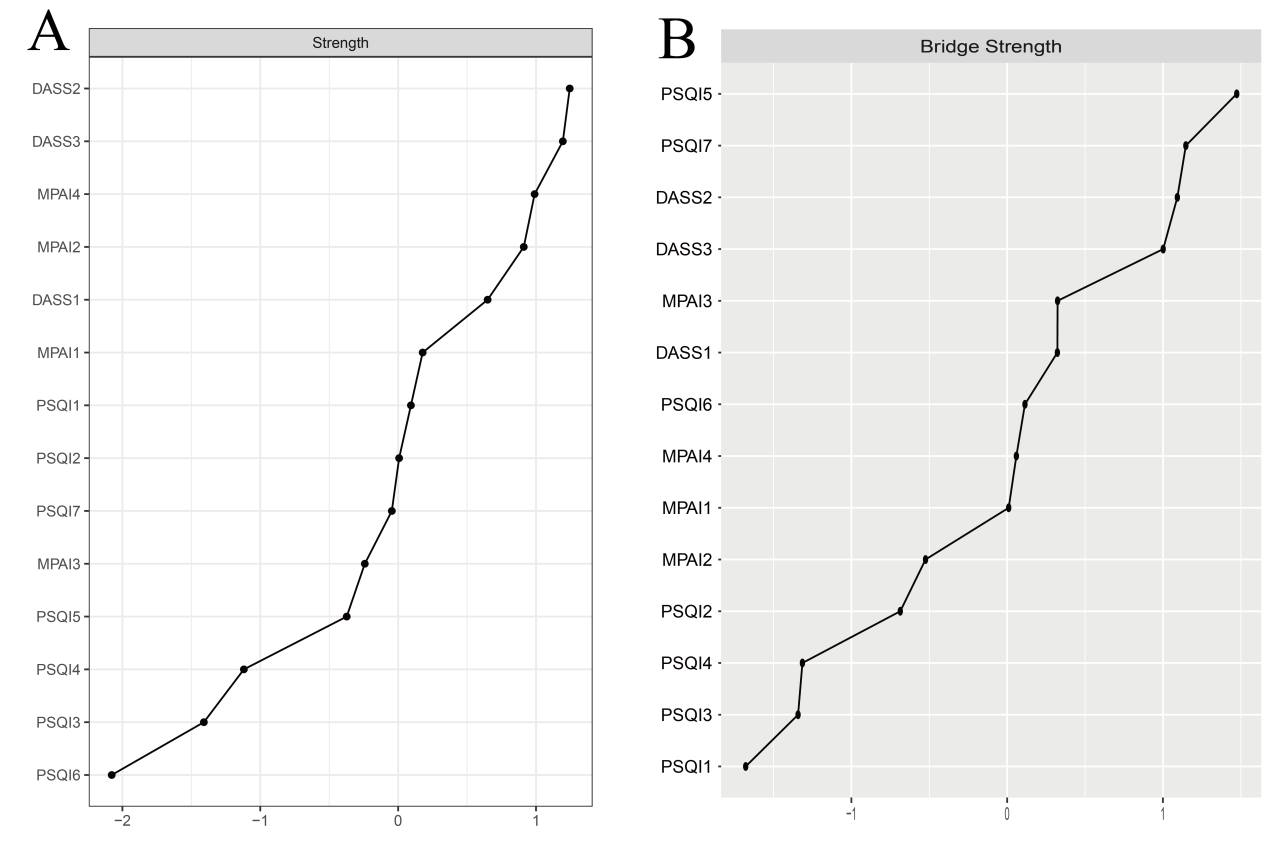


**Figure S7.** Standardized strength and bridge strength centrality indices for the integrated DASS-PSQI-MPAI dimension-/component-level network among the study participants (z-scores). (A) Strength centrality; (B) bridge strength centrality.


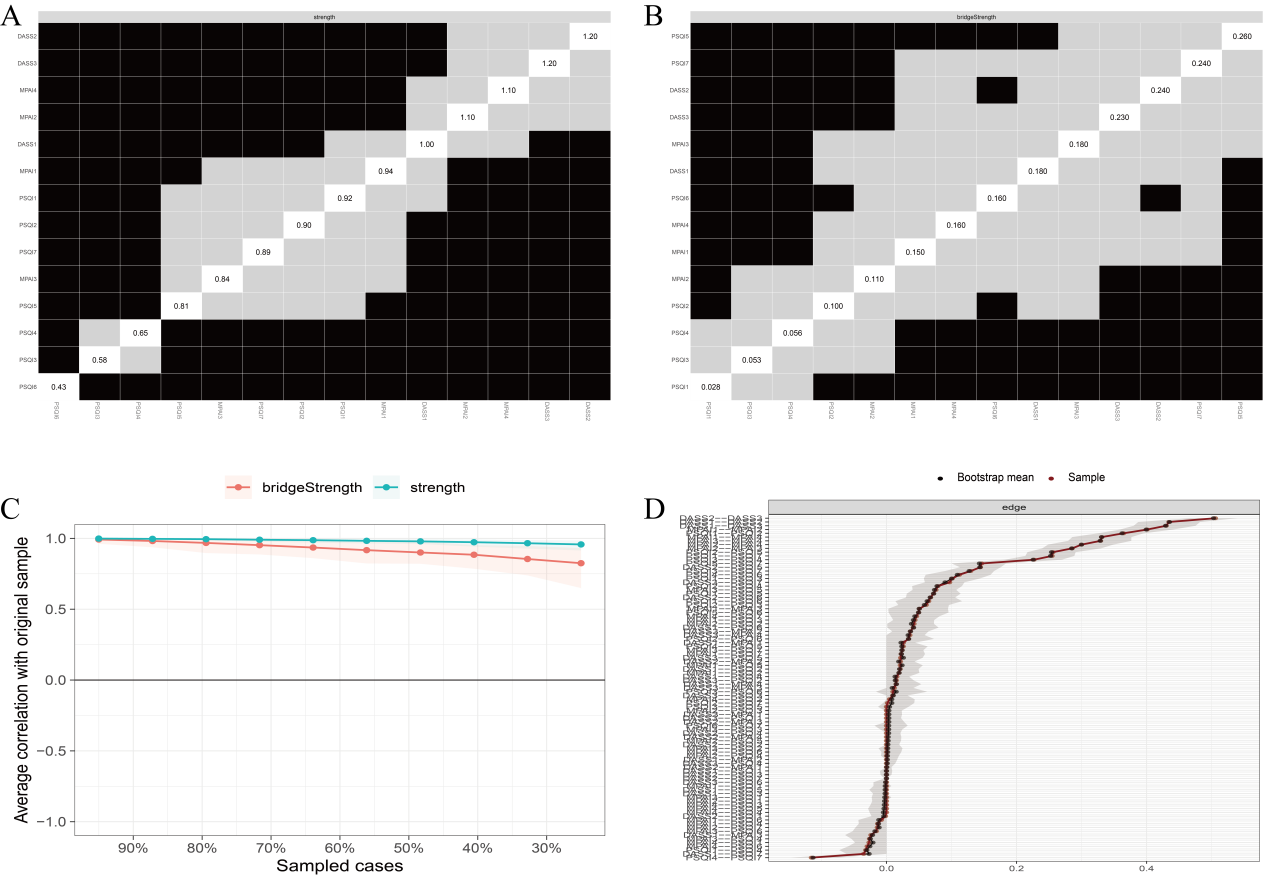


**Figure S8.** Stability and accuracy assessment of the integrated DASS-PSQI-MPAI dimension-/component-level network among the study participants. (A) Bootstrap difference tests for nodal strength centrality. (B) Bootstrap difference tests for bridge strength centrality. Gray cells indicate non-significant differences, black cells indicate significant differences, and diagonal white cells display the original sample estimates. (C) Case-dropping bootstrap analysis of the stability of strength and bridge strength centrality. (D) Bootstrapped 95% confidence intervals for edge weights. All bootstrap analyses were based on 1,000 bootstrap samples.

**Table S1.** Mapping between network nodes and corresponding scale dimensions, components, or items.

| **Scale** | **Abbr** | **Symptoms**  **/Dimensions** | **Items** |
| --- | --- | --- | --- |
| DASS | DASS1 | Depression symptoms | 3. I could not seem to experience any positive feeling at all. |
|  |  |  | 5. I found it difficult to work up the initiative to do things. |
|  |  |  | 10. I felt that I had nothing to look forward to. |
|  |  |  | 13. I felt down-hearted and blue. |
|  |  |  | 16. I was unable to become enthusiastic about anything. |
|  |  |  | 17. I felt I was not worth much as a person. |
|  |  |  | 21. I felt that life was meaningless. |
|  | DASS2 | Anxiety symptoms | 2. I was aware of dryness of my mouth. |
|  |  |  | 4. I experienced breathing difficulty (e.g., excessively rapid breathing, breathlessness in the absence of physical exertion). |
|  |  |  | 7. I experienced trembling (e.g., in the hands). |
|  |  |  | 9. I was worried about situations in which I might panic and make a fool of myself. |
|  |  |  | 15. I felt I was close to panic. |
|  |  |  | 19. I was aware of the action of my heart in the absence of physical exertion (e.g., sense of heart rate increase, heart missing a beat). |
|  |  |  | 20. I felt scared without any good reason. |
|  | DASS3 | Stress symptoms | 1. I found it hard to wind down. |
|  |  |  | 6. I tended to over-react to situations. |
|  |  |  | 8. I felt that I was using a lot of nervous energy. |
|  |  |  | 11. I found myself getting agitated. |
|  |  |  | 12. I found it difficult to relax. |
|  |  |  | 14. I was intolerant of anything that kept me from getting on with what I was doing. |
|  |  |  | 18. I found that I was rather touchy. |
| PSQI | PSQI1 | Sleep Quality | 6. During the past month, how would you rate your sleep quality overall? |
|  | PSQI2 | Sleep Latency | 2. During the past month, how long (in minutes) has it usually taken you to fall asleep each night? |
|  |  |  | 5a. During the past month, cannot get to sleep within 30 minutes. |
|  | PSQI3 | Sleep Duration | 4. During the past month, how many hours of actual sleep did you get at night? |
|  | PSQI4 | Sleep Efficiency | 1. During the past month, when have you usually gone to bed at night? |
|  |  |  | 2. During the past month, how long (in minutes) has it usually taken you to fall asleep each night? |
|  |  |  | 4. During the past month, how many hours of actual sleep did you get at night? |
|  | PSQI5 | Sleep Disturbance | 5b. Wake up in the middle of the night or early morning. |
|  |  |  | 5c. Have to get up to use the bathroom. |
|  |  |  | 5d. Cannot breathe comfortably. |
|  |  |  | 5e. Cough or snore loudly. |
|  |  |  | 5f. Feel too cold. |
|  |  |  | 5g. Feel too hot. |
|  |  |  | 5h. Have bad dreams. |
|  |  |  | 5i. Have pain. |
|  |  |  | 5j. Other reasons disturbing sleep. |
|  | PSQI6 | Hypnotic Medication | 7. During the past month, how often have you taken medicine to help you sleep? |
|  | PSQI7 | Daytime Dysfunction | 8. During the past month, how often have you had trouble staying awake while driving, eating meals, or engaging in social activity? |
|  |  |  | 9. During the past month, how much of a problem has it been for you to keep up enough enthusiasm to get things done? |
| MPAI | MPAI1 | Loss of Control | 1. Your friends and family have complained about you using your phone. |
|  |  |  | 2. Someone has said you’ve spent too much time on your phone. |
|  |  |  | 3. You have tried to hide how long you spend on your phone from others. |
|  |  |  | 4. You have exceeded your phone bill. |
|  |  |  | 5. You find yourself using your phone longer than you intended. |
|  |  |  | 6. You have tried to spend less time on your phone but haven’t been able to. |
|  |  |  | 7. You never feel like you spend enough time on your phone. |
|  | MPAI2 | Withdrawal | 8. When you’re out of mobile service areas for a while, you always worry about missing calls. |
|  |  |  | 9. You find it difficult to turn your phone off. |
|  |  |  | 10. If you haven’t checked your messages or turned on your phone for a while, you feel anxious. |
|  |  |  | 11. You feel restless without your phone. |
|  | MPAI3 | Escapism | 12. If you don’t have your phone, your friends have a hard time reaching you. |
|  |  |  | 13. When feeling isolated, you chat with others using your phone. |
|  |  |  | 14. When you feel lonely, you chat with others using your phone. |
|  | MPAI4 | Inefficiency | 15. When feeling down, you play with your phone to change your mood. |
|  |  |  | 16. You find yourself engrossed in your phone even when you have other important tasks, which causes you trouble. |
|  |  |  | 17. The time spent on your phone directly reduces your work efficiency. |

**Table S2a**. Descriptive statistics, centrality indices, bridge strength, and node predictability in the DASS-MPAI dimension-level network.

| **Variable** | **Label** | **Mean** | **SD** | **Skewness** | **Kurtosis** | **Strength** | **Bridge strength** | **Predictability (R2)** |
| --- | --- | --- | --- | --- | --- | --- | --- | --- |
| DASS1 | Depression | 3.416 | 4.176 | 1.484 | 2.457 | 0.945 | 0.068 | 0.879 |
| DASS2 | Anxiety | 3.610 | 4.134 | 1.390 | 2.120 | 1.011 | 0.038 | 0.895 |
| DASS3 | Stress | 3.924 | 4.362 | 1.157 | 1.276 | 1.063 | 0.105 | 0.896 |
| MPAI1 | Loss of control | 16.012 | 6.087 | 0.325 | -0.183 | 0.832 | 0.037 | 0.703 |
| MPAI2 | Withdrawal | 8.822 | 3.838 | 0.442 | -0.397 | 1.056 | 0.064 | 0.756 |
| MPAI3 | Escapism | 7.309 | 3.015 | 0.272 | -0.449 | 0.735 | 0.058 | 0.624 |
| MPAI4 | Inefficiency | 6.941 | 2.994 | 0.365 | -0.411 | 1.018 | 0.051 | 0.743 |

**Table S2b.** Descriptive statistics, centrality indices, bridge strength, and node predictability in the PSQI-MPAI component-/dimension-level network.

| **Variable** | **Label** | **Mean** | **SD** | **Skewness** | **Kurtosis** | **Strength** | **Bridge strength** | **Predictability (R2)** |
| --- | --- | --- | --- | --- | --- | --- | --- | --- |
| MPAI1 | Loss of control | 16.012 | 6.087 | 0.325 | -0.183 | 0.951 | 0.156 | 0.705 |
| MPAI2 | Withdrawal | 8.822 | 3.838 | 0.442 | -0.397 | 1.125 | 0.113 | 0.754 |
| MPAI3 | Escapism | 7.309 | 3.015 | 0.272 | -0.449 | 0.816 | 0.155 | 0.625 |
| MPAI4 | Inefficiency | 6.941 | 2.994 | 0.365 | -0.411 | 1.109 | 0.120 | 0.740 |
| PSQI1 | Sleep quality | 0.863 | 0.705 | 0.411 | -0.216 | 0.929 | 0.028 | 0.371 |
| PSQI2 | Sleep latency | 0.922 | 0.853 | 0.624 | -0.326 | 0.957 | 0.087 | 0.424 |
| PSQI3 | Sleep duration | 0.798 | 0.692 | 0.578 | 0.255 | 0.597 | 0.049 | 0.167 |
| PSQI4 | Sleep efficiency | 0.714 | 0.820 | 0.915 | 0.042 | 0.655 | 0.034 | 0.107 |
| PSQI5 | Sleep disturbance | 0.828 | 0.670 | 0.447 | 0.122 | 0.949 | 0.189 | 0.410 |
| PSQI6 | Hypnotic medication | 0.121 | 0.450 | 4.191 | 18.466 | 0.455 | 0.042 | 0.107 |
| PSQI7 | Daytime dysfunction | 1.169 | 0.961 | 0.280 | -0.975 | 0.860 | 0.114 | 0.319 |

**Table S2c.** Descriptive statistics, centrality indices, bridge strength, and node predictability in the integrated DASS-PSQI-MPAI dimension-/component-level network.

| **Variable** | **Label** | **Mean** | **SD** | **Skewness** | **Kurtosis** | **Strength** | **Bridge strength** | **Predictability (R2)** |
| --- | --- | --- | --- | --- | --- | --- | --- | --- |
| DASS1 | Depression | 3.416 | 4.176 | 1.484 | 2.457 | 1.042 | 0.178 | 0.881 |
| DASS2 | Anxiety | 3.610 | 4.134 | 1.390 | 2.120 | 1.175 | 0.235 | 0.899 |
| DASS3 | Stress | 3.924 | 4.362 | 1.157 | 1.276 | 1.164 | 0.228 | 0.899 |
| MPAI1 | Loss of control | 16.012 | 6.087 | 0.325 | -0.183 | 0.937 | 0.154 | 0.706 |
| MPAI2 | Withdrawal | 8.822 | 3.838 | 0.442 | -0.397 | 1.101 | 0.114 | 0.757 |
| MPAI3 | Escapism | 7.309 | 3.015 | 0.272 | -0.449 | 0.843 | 0.178 | 0.629 |
| MPAI4 | Inefficiency | 6.941 | 2.994 | 0.365 | -0.411 | 1.118 | 0.158 | 0.745 |
| PSQI1 | Sleep quality | 0.863 | 0.705 | 0.411 | -0.216 | 0.918 | 0.028 | 0.371 |
| PSQI2 | Sleep latency | 0.922 | 0.853 | 0.624 | -0.326 | 0.899 | 0.102 | 0.427 |
| PSQI3 | Sleep duration | 0.798 | 0.692 | 0.578 | 0.255 | 0.582 | 0.053 | 0.167 |
| PSQI4 | Sleep efficiency | 0.714 | 0.820 | 0.915 | 0.042 | 0.647 | 0.056 | 0.109 |
| PSQI5 | Sleep disturbance | 0.828 | 0.670 | 0.447 | 0.122 | 0.814 | 0.264 | 0.488 |
| PSQI6 | Hypnotic medication | 0.121 | 0.450 | 4.191 | 18.466 | 0.432 | 0.162 | 0.156 |
| PSQI7 | Daytime dysfunction | 1.169 | 0.961 | 0.280 | -0.975 | 0.887 | 0.239 | 0.339 |

**Table S3.** Zero-order correlation matrix of DASS dimensions, MPAI dimensions, and PSQI components.

|  | **DASS1** | **DASS2** | **DASS3** | **MPAI1** | **MPAI2** | **MPAI3** | **MPAI4** | **PSQI1** | **PSQI2** | **PSQI3** | **PSQI4** | **PSQI5** | **PSQI6** | **PSQI7** |
| --- | --- | --- | --- | --- | --- | --- | --- | --- | --- | --- | --- | --- | --- | --- |
| **DASS1** | 0.00 |  |  |  |  |  |  |  |  |  |  |  |  |  |
| **DASS2** | 0.43 | 0.00 |  |  |  |  |  |  |  |  |  |  |  |  |
| **DASS3** | 0.43 | 0.51 | 0.00 |  |  |  |  |  |  |  |  |  |  |  |
| **MPAI1** | 0.03 | 0.00 | 0.00 | 0.00 |  |  |  |  |  |  |  |  |  |  |
| **MPAI2** | 0.00 | 0.02 | 0.04 | 0.40 | 0.00 |  |  |  |  |  |  |  |  |  |
| **MPAI3** | -0.02 | 0.00 | 0.01 | 0.05 | 0.29 | 0.00 |  |  |  |  |  |  |  |  |
| **MPAI4** | 0.01 | 0.00 | 0.04 | 0.33 | 0.30 | 0.33 | 0.00 |  |  |  |  |  |  |  |
| **PSQI1** | 0.00 | -0.00 | 0.00 | 0.00 | 0.00 | 0.00 | -0.03 | 0.00 |  |  |  |  |  |  |
| **PSQI2** | 0.02 | 0.00 | 0.02 | 0.02 | 0.04 | 0.00 | 0.00 | 0.36 | 0.00 |  |  |  |  |  |
| **PSQI3** | 0.00 | 0.00 | 0.01 | 0.04 | 0.00 | 0.00 | 0.00 | 0.10 | 0.06 | 0.00 |  |  |  |  |
| **PSQI4** | 0.02 | 0.00 | 0.00 | -0.01 | 0.00 | -0.03 | 0.00 | -0.03 | 0.08 | 0.23 | 0.00 |  |  |  |
| **PSQI5** | 0.00 | 0.14 | 0.02 | 0.02 | 0.00 | 0.08 | 0.00 | 0.07 | 0.26 | 0.00 | 0.03 | 0.00 |  |  |
| **PSQI6** | 0.04 | 0.07 | 0.00 | -0.01 | 0.00 | -0.02 | -0.03 | 0.06 | 0.03 | 0.01 | 0.11 | 0.05 | 0.00 |  |
| **PSQI7** | -0.04 | 0.00 | 0.10 | 0.02 | -0.01 | 0.02 | 0.05 | 0.25 | 0.00 | 0.13 | -0.12 | 0.15 | 0.00 | 0.00 |

**Table S4.** Comparison of MPAI node strength and bridge strength across the two two-domain networks.

| **MPAI node** | **Strength in DASS-MPAI** | **Strength in PSQI-MPAI** | **Bridge strength in DASS-MPAI** | **Bridge strength in PSQI-MPAI** |
| --- | --- | --- | --- | --- |
| MPAI1 Loss of control | 0.832 | 0.951 | 0.037 | 0.156 |
| MPAI2 Withdrawal | 1.056 | 1.125 | 0.064 | 0.113 |
| MPAI3 Escapism | 0.735 | 0.816 | 0.058 | 0.155 |
| MPAI4 Inefficiency | 1.018 | 1.109 | 0.051 | 0.120 |
